# Case-finding for alpha1-antitrypsin deficiency in Kazakh patients with COPD

**DOI:** 10.1186/s40248-017-0104-5

**Published:** 2017-10-25

**Authors:** Ardak Zhumagaliyeva, Stefania Ottaviani, Timm Greulich, Marina Gorrini, Claus Vogelmeier, Ludmila Karazhanova, Gulmira Nurgazina, Annalisa DeSilvestri, Victor Kotke, Valentina Barzon, Michele Zorzetto, Angelo Corsico, Ilaria Ferrarotti

**Affiliations:** 1 Semey State Medical University, Semey, Kazakhstan; 2 Center for Diagnosis of Inherited Alpha1-antitrypsin Deficiency, Dept of Internal Medicine and Therapeutics Pneumology Unit, IRCCS San Matteo Hospital Foundation University of Pavia, Piazza Golgi 1, 27100 Pavia, Italy; 3 University Clinic of Marburg and Gissen, Center for Research alpha-1-antitrypsin deficiency, Marburg, Germany; 4 Dept of Internal Medicine and Therapeutics, Pneumology Unit, University of Pavia, Pavia, Italy; 5 Kazakh Medical University of Continuing Education, Almaty, Kazakhstan; 6 Statistics Department Fondazione IRCCS Policlinico San Matteo, Pavia, Italy

**Keywords:** Alpha-1 antitrypsin, Chronic obstructive pulmonary disease, Genetics, Genotype, Orphan disease

## Abstract

**Background:**

Alpha-1-antitrypsin deficiency (AATD) is an under-diagnosed condition in patients with chronic obstructive pulmonary disease (COPD). The aim of this study was to screen for AATD in Kazakh patients with COPD using dried blood spot specimens.

**Methods:**

The alpha1-antitrypsin (AAT) concentration was determined by nephelometry, PCR was used to detect PiS and PiZ alleles; and isoelectric focusing was used to confirm questionable genotype results and detect rare AAT variants.

**Results:**

To this aim, 187 Kazakh subjects with COPD were recruited. Blood samples were collected as dried blood spot. Genotyping of 187 samples revealed 3 (1.6%) PI*MZ and 1 (0.53%) PI*MS, Phenotyping identified also two sample (1.1%) with phenotype PiMI. Allelic frequencies of pathological mutations Z, S and I resulted 0.8%, 0.3%, 0.5%, respectively, in COPD Kazakh population.

**Conclusion:**

This study proved that AATD is present in the Kazakh population. These results support the general concept of targeted screening for AAT deficiency in countries like Kazakhstan, with a large population of COPD patients and low awareness among care-givers about this genetic condition.

## Background

Chronic obstructive pulmonary disease (COPD) is a principal cause of morbidity and mortality. In 2008, COPD was ranked fourth as a leading cause of death worldwide, and the number of patients is still increasing. The World Health Organization (WHO) predicts that COPD will reach third among the most common causes of mortality by 2030 [1]. In Kazakhstan, the number of patients with COPD has increased more than twofold in the last 10 years, with an incidence of 321 out of 100 thousand people in 2011 [2].

Alpha1-antitrypsin deficiency (AATD), the most widely recognized genetic disorder causing COPD [3, 4] was first reported in 1963 by Carl-Bertil Laurell and his fellow investigator Sten Eriksson, who detected the lack of a normal alpha1-band on plasma protein electrophoresis in two emphysematous patients [5]. After this initial discovery, over 100 variants of alpha 1-antitrypsin (AAT) have been detected; these have been characterized as different genetic variants. Subsequent studies on the prevalence of these variants have greatly contributed to our understanding of the epidemiology of the disorder.

Alpha1-antitrypsin is a 52-kDa glycoprotein produced by hepatocytes and, to a lesser extent, by mononuclear monocytes. Its main function is to protect the lung against proteolytic damage by neutrophil elastase [6]. The AAT protein is encoded by the *SERPINA1* gene, which is situated on the long arm of chromosome 14 (14q31–32.3). This gene spans 12.2 kb and is organized into four coding (II, III, IV and V) and three non-coding (Ia, Ib and Ic) exons. The encoded protein includes 394 amino acids, with its reactive center loop corresponding to methionine-358 [7]. AAT inhibits several serine proteinases, but its preferred target is neuthrophil elastase (NE), a 29- kDa neutrophil enzyme that facilitates elastin degradation and lung tissue injury and destruction. When neutrophils are activated, preformed NE is secreted into the lung tissue. Normal AAT plasma levels (1–2 g/L) protect lungs from NE attack, binding the enzyme to the AAT active site and constantly inactivating the enzyme [8]. The pathophysiology of AAT is associated with mutations in the PI locus [9]. The most common deficiency allele is the Z allele (rs28929474) which, in the homozygous state (PiZZ), is associated with AAT plasma levels that are 85% less than normal. The S allele (rs17580) is associated with AAT plasma levels that are nearly 40% less than normal in the homozygous state [10]. The normal allele, usually called M, is characterized by an AAT plasma level that falls within general population normal ranges.

Epidemiological studies have shown that the highest prevalence of PI*ZZ related AATD is among Northern Europeans and populations with a Northern European background (8). Nevertheless, during the last few years, based on evaluations of allele frequencies in available cohort studies, it has been suggested that the Z variant is not only common in Caucasians, but also among other ethnic groups worldwide [11, 12]. Furthermore, there are at least 30 AAT alleles other than the PI*Z and PI*S alleles which are associated with significantly reduced or absent plasma AAT levels. Given the extreme rarity of such variants, often described in the literature as single case reports, little is known about their epidemiology, especially in countries where this disorder is largely under-diagnosed [13].

As an under-diagnosed disorder, the latest AATD guidelines by both the World Health Organization and the American Thoracic Society/European Respiratory Society recommend the establishment of screening programs to detect AATD in patients with COPD. It is rationalized that the coincidental identification of AATD would motivate family screening, while improving appropriate management, and specific counseling for these patients and families [14].

Even though AATD is as a whole, one of the most common hereditary disorders worldwide, its frequency varies markedly from one country to another [15–17] and affects many different racial subgroups. In particular, AATD has spread significantly throughout the continent of Asia [18]. Therefore, with this study we aimed to detect AAT deficiency in Kazakh COPD patients.

## Methods

### Study subjects

After approval by the local Ethical Committee (№2, 13.11.2013), all compliant COPD patients referred to the Pulmonary Disease Department in Emergency Hospital of Semey (East Kazakhstan region) from June 2014 to August 2014 and from July 2015 to September 2015 were enrolled in the present study. The analysis was conducted in two major European reference centers for AATD: Centre for research alpha-1-antitrypsin deficiency, Marburg, Germany and Center for Diagnosis of Inherited Alpha1-antitrypsin deficiency, Institute for Respiratory Disease, Pavia, Italy.

According to the protocol: Global Initiative for Chronic Obstructive Lung Disease (GOLD) [19], COPD diagnosis was confirmed by spirometry with FEV_1_/FVC values <0.7, where FEV_1_ = forced expiratory volume in one second and FVC = forced vital capacity. Pre-bronchodilator spirometric tests were performed according to ERS guidelines with a rolling seal spirometer followed by post bronchodilator spirometric tests after inhalation of 400 mg salbutamol [20]. After a targeted physical examination, data on patient symptoms were collected. Smoking history was calculated in pack/years as the product of tobacco use (in years) and the average number of cigarettes smoked per day/20 [21].

Capillary blood samples were collected on filter paper. Blood was obtained by pricking a distal finger tip and blotting onto filter paper. The papers were then air-dried at room temperature and stored at 4 °C in separate envelopes to avoid cross contamination.

### Quantitative determination of AAT level

The AAT level measurements were performed on dried blood spot (DBS) samples by a rate immune nephelometric method (Dade-Behring BN II, Germany and Immage 800 Immunochemistry System - (Beckman-Coulter, USA) [9, 22].

### Genotyping and phenotyping

DNA extracted from the DBS of all subjects by standard methods was submitted to genotyping for Z and S *SERPINA1* alleles [23, 24]. Qualitative detection and characterization of AAT phenotypes was carried out by IsoElectroFocusing (IEF) using the Hydrasys electrophoresis platform and the Hydragel 18 AAT Isofocusing kit ​(Sebia, Spain) [25].

### Statistical analysis

Data were analyzed using the Statistical Package for the Social Sciences version 20, (SPSS, USA, Chicago, IL). Descriptive statistics were used to analyse data. For continuous variables, the mean, standard deviation (SD), median, minimum and maximum values were calculated.

## Results

**Fig. 1:**
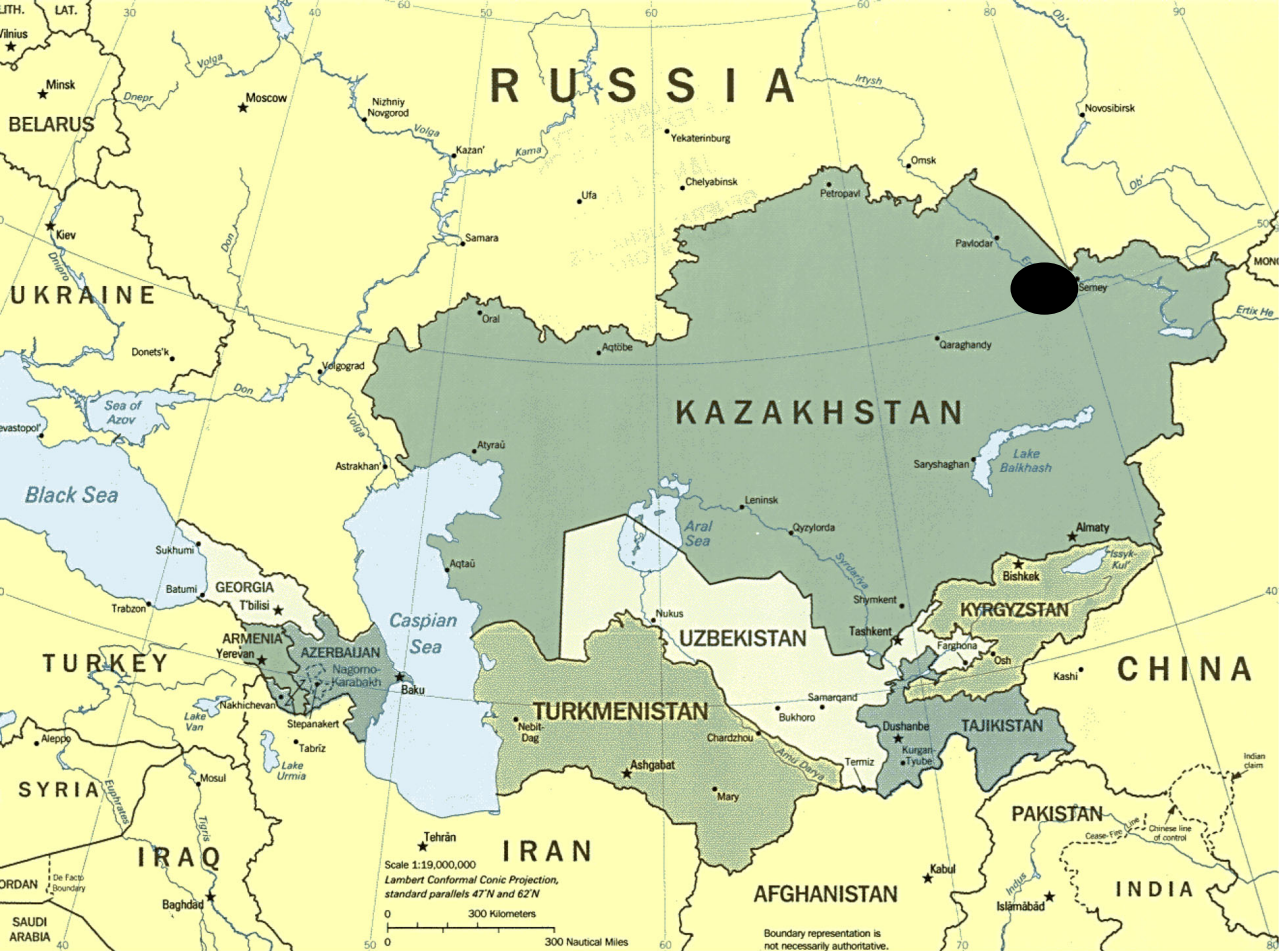
Geographic map of Kazakhstan. The black circle indicates the area of Semey, where the COPD patients object of the present study have been recruited

One hundred eleven subjects with COPD (group 1) were enrolled during the period from June to August 2014 and analysed in Germany. Among those, 64 were male and 47 female, the mean age was 60.7 years (SD 11.3; range 21–79). The large majority of patients were non-smokers (54.05%), current and former smokers were 34.2% and 10.8% respectively. Seventy-six Kazakh subjects (26 male, 50 female) with COPD (group 2) were enrolled during the period from July to September – 2015 and analyzed in Italy. Their mean age was 57.7 years (SD 13.03; range 24–79). The percentage of non-smokers was 64.5%, current and former smokers were 22.4% and 10.8% respectively. Table 1 summarizes the characteristics of the two groups of samples. No statistically significant demographic or clinical variations were revealed between the two groups. All the COPD patients belonged to the Semey area in the north-east of Kazakhstan (Fig. 1).

**Table 1:** Patient demographic characteristics, spirometric values and clinical data

	Group 1	Group 2
*N*	111	76
Age (years) ÷ mean (SD)	60.7(11.3)	57.98(11.03)
Male %	57.6	34.2
BMI (kg/m^2^) ÷ mean (SD)	22.14(4,33)	21.92(3,8)
FEV_1_/FVC^a^	0.56(0.16–0.68)	0.57 (0.26–0.69)
FEV_1_% Predicted^a^	65(18–78)	65(25–77)
FVC (%)^a^	66(12)(1.66–5,12)	60(11,9)(1.66–5.12)
MRC (score)^a^	2(0–4)	2(0–4)
CAT (score)^a^	28(4–40)	15(5–40)
Current/former/non smoker (%)	34.2/10.8/54.05	22.4/10.8/64.5
Pack/year÷ (years) mean (SD)	14.48(23.75)	8.78(13.48)

*SD* Standard deviation; ^a^median, lowest value and highest value; *MRC* Medical research council scale, CAT- COPD assessment test

**Fig. 2:**
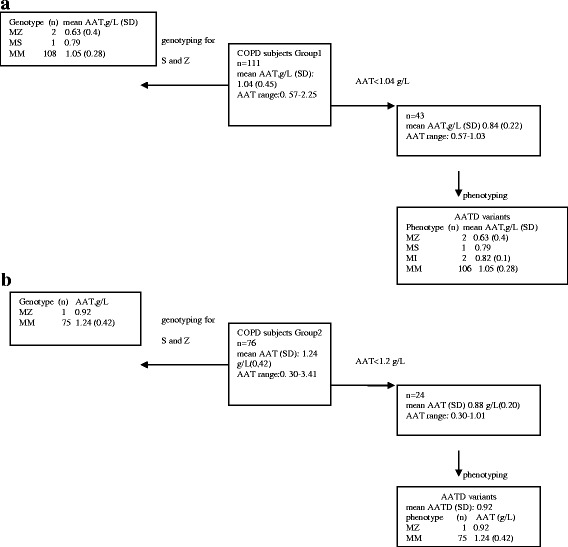
Schematic representation of the genotyping/phenotyping results in groups 1 (**a**) and 2 (**b**)

Mean AAT plasma concentrations were 1.04 g/l (SD 0.45; range 0.57–2.25) and 1.24 g/l (SD 0.42; range 0.30–3.41) in groups 1 and 2 respectively. S and Z allele genotyping allowed the detection of two PI*MZ, and one PI*MS in group 1 and one PI*MZ in group 2. By applying the cut - off concentrations used in the German and Italian Centers (1.04 g/L and 1.2 g/L, respectively), we performed IEF [25] in 43 samples from group 1 and 24 samples from group 2 (Fig. 2).

The phenotyping analysis of these samples, confirmed the genotyping results for samples carrying a PI*MZ or PI*MS genotype and revealed two subjects with a PIMI phenotype in group 1 (Fig. 1). Genotype and allele frequencies are reported in Table 2. Demographic and clinical data of patients with AAT deficiency, as established by genotyping and phenotyping are reported in Table 3.

**Table 2:** Frequency of AAT genotypes and alleles in the study population

	Group 1 (%)	Group 2 (%)	Total (%)
PI*MM	95.5	98.7	96.8
PI*MS	0.9	-	0.5
PI*MZ	1.8	1.3	1.6
PI MI	1.8	-	1.1
M allele	97.7	99.3	98.4
S allele	0.4	-	0.3
Z allele	0.9	0.7	0.8
I allele	0.9	-	0.6

**Table 3:** Demographic and clinical data of patients with AAT deficiency

	PIMZ	PIMS	PIMI	Total
*N*	3	1	2	6
Age (years) ÷ mean (SD)	59(16.9)	62	51(2)	56.8(12.7)
Male %	33.3	100	50	50
BMI (kg/m^2^) ÷ mean (SD)	22.7(0,9)	18	25(2.5)	21.2(2.3)
FEV_1_/FVC ÷ mean (SD)	0.48(0.2)	0.34	0.48(0.02)	0.47(0.6)
FEV_1_% Predicted ÷ mean (SD)	46.5(2.1)	40	48.5(0.7)	48(4.5)
FVC(%) ÷ mean (SD)	51.3(4.05)	47	49.5(3.5)	2.9(0.2)
MRC (score) ÷ mean (SD)	2	3	2	2.3 (0.5)
CAT (score) ÷ mean (SD)	29.5(2.1)	40	34.5(6.3)	34.3 (9.9)
Current/former/non smoker (%)	0/33,3/0	0/0/0	50/0/0	16,6/16,6/0
Pack/year ÷ (years) mean (SD)	20	0	34	27(7)

## Discussion

In Asian populations a systematic review of published data showed that *SERPINA1* PI*S and PI*Z deficiency alleles are very rare, as also recently reported by de Serres and Blanco [26] in a genetic epidemiology study investigating AAT deficiency in the major geographic regions worldwide. Although this comprehensive study has potential biases, especially in regions with different ethnic or racial groups, it clearly pointed out the low frequency of S and Z alleles in Northern and Central Asian countries, with the exception of Saudi Arabia. According to the study, PI*S frequencies range from 0.0 in Nepal and Kazakhstan to 31 per 1000 inhabitants in Saudi Arabia; whereas, PI*Z frequencies range from less than 1 in Nepal, India, Jordan and Israel to 15 in Saudi Arabia.

Studies on AAT deficiency as a cause of COPD have only rarely been performed in Asian countries. A study on 356 COPD patients and 185 healthy controls demonstrated a higher frequency for S and F alleles in the COPD group compared with controls, although their frequencies were very low (0.017 and 0.014 respectively) [27]. In China, electrophoretic analysis of 748 normal subjects and 414 COPD patients did not detect any S or Z mutations in either COPD group [28], nor were any S or Z variants identified in a Korean study on 56 male emphysematous patients over 50 years old [29]. Likewise, in Iran, an investigation of 130 COPD patients detected no S and Z variants [30]. While in India, genotyping of 200 COPD patients identified two SS (1%) and one ZZ patient (0.5%) [31].

On the other hand, an investigation of 158 healthy subjects in Saudi Arabia showed that 2.53% were heterozygous for the Z mutation, 11.39% were heterozygous for the S mutation and 3.8% had an SZ genotype [32]. The high frequency in Saudi Arabia could be explained by movement of people over time to major cities in that country [12].

Kazakhstan is a Central Asian country, which has historically been inhabited by nomadic tribes. Indeed, the indigenous population is made up of Kazakhs, a Turkish-speaking people. Anthropologically, Kazakhs belong to the Southern Siberian lineage, the majority of whom have racially-diagnostic features intermediate between Caucasians and Mongoloids, with some predominant Mongoloid components [33, 34].

Recent investigations in the Kazakh population confirmed a remarkable frequency of AATD variants in the general population and healthy subjects. The IEF method in an ultra-thin polyacrylamide gel identified variants of alpha1-antitrypsin in 218 indigenous residents from three ethno-historical regions of the Kazakh Soviet Socialist Republic. The frequencies of alleles - PIM1, PIM2, PIM3 - in the total sample were 0.8477, 0.1372 and 0.0106, respectively, the overall frequency of rare phenotypes (PIN and PIZ) was 0.0046 [35], which however resulted smaller than the frequency of rare pathological genotypes detected in the present paper (0.032). This discrepancy could mainly be explained by the more sensible and updated diagnostic approach we used.

An intergroup variability analysis of phenotypes and allele frequencies of alpha1-antitrypsin showed a clear local diversity. PIM1 frequency among Kazakh residents of the Northern-central area was significantly lower, while the PIM2 frequency was significantly higher than in the Southeastern and Western regions. Accordingly, the PIM3 frequency in the sample did not differ. The results were compared with the published data on alpha-1 antitrypsin polymorphisms in Eurasian populations. The PIM1 and PIM2 allele frequencies in Kazakhs differed from the corresponding average values ​​for both Caucasian and Mongoloid groups. However, they were intermediate between Caucasian and Mongoloid frequency estimates, as might be expected considering the mixed origin of the Kazakh people and the Kazakhstan geographical position and its boundary between Europe and Asia [34].

The present screening of COPD Kazakh patients showed that Z and S allele frequencies (although low) were not that different from other studies on Caucasian populations. The percentage of PI*MZ cases (1.6%) detected was half that of the Spanish study performed in the primary care setting in COPD patients. In that study, nineteen patients out of 596 (3.2%) were carriers of the allelic variant Z, two of whom were homozygous for PiZZ and one heterozygous for PiSZ [36].

In this study, we extended the *SERPINA1* gene investigation to variants not detectable by PI*S and PI*Z genotyping. With this strategy, we found two samples with the rare deficiency variant I (frequency 1.1%). The I mutation occurs at the residue, arginine 39, which is involved in the formation of an ionic bond with glutamic acid 264 [37]. Although no subjects with severe AATD deficiency were detected in this study, we found six out of 187 (5%) subjects positive for so called “intermediate genetic AAT deficiency” [mean (SD) AAT level: 0.85 g/L (0.17)]. These findings are consistent with the hypothesis that intermediate AATD, such as PI*MZ and PI*MI, represents a risk factor for developing COPD [38, 39]. An interesting meta-analysis [40] reported that the increased risk for COPD in PI*MZ heterozygous individuals (OR for PI*MZ versus PI*MM (normal genotype) was 2.31 (95% CI 1.60 to 3.35). These data underscore the importance of genetic screening for AATD in Kazakhstan, as it permits the identification of a rarely identified disorder.

We compared our data with other studies on patients with COPD. The largest percentage (4.2%) of rare mutations was found in the study by Sabri et al. on Tunisian patients with COPD. In our study, the frequency of the rare mutation PIMI was 1.1% (Table 4). To characterize the genetic origin of the Kazakhs in relation to Europeans and Mongoloids, the polymorphisms of *SERPINA1* gene need to be studied among the peoples of Tunguss, Turkic and Mongol origin, whose ancestors participated in the formation of the South Siberian anthropological Kazakh [33, 41]. On the contrary, the analysis of people with Chinese origin could have less importance regarding this goal, since the Chinese were not nomads and moved to already conquered countries. Thus, past population migrations appears to be the most reasonable explanation for transport of deficiency alleles from Europe to Kazakhstan.

**Table 4:** Comparison of the current study with data on COPD cohorts from the literature

Phenotype Studies (*n* = number of patients)	Normal phenotype	Alpha-1-antitrypsin mutation Phenotypes						Inclusion criteria
	PiMM *n* %	PiMZ *n* %	PiMS *n* %	PiSS *n* %	PiSZ *n* %	PiZZ *n* %	Other phenotypes *n* %	
Mittman et al. 1974 [43] (*n* = 240) USA	194 80,8%	20 8,4%	17 7,1%	1 0,4%	2 0,8%	6 2,5%	0 0%	Chronic bronchitis and/or emphysema
Cox et al. 1976 [44] (*n* = 163) USA	139 85,3%	8 4,9%	7 4,3%	0 0%	0 0%	8 4,9%	1 0,6%	COPD and aged over 18 years
Liebermann et al. 1986 [45] (*n* = 965) USA	not indicated	74 7,7%	86 8,9%	3 0,3%	3 0,3%	18 1,9%	not indicated	COPD
Sitkauskiene et al. 2008 [46] (*n* = 1167) Lithuania	not indicated	40 3,4%	39 3,3%	1 0,1%	3 0,3%	8 0,7%	0 0%	COPD according to GOLD
Sabri Denden et al. 2008 [47] (*n* = 120) Tunisia	114 95%	1 0.83%	0 0%	0 0%	0 0%	0 0%	5 4,2%	COPD according to GOLD
Molina et al. [36] 2009 (*n* = 596) Spain	487 81,7%	16 2,7%	80 13,4%	10 1,7%	1 0,2%	2 0,3%	0 0%	COPD according to GOLD
Novak T et al. 2011, [48] (*n* = 105) Germany	94 89,5%	4 3,8%	6 5,7%	0 0%	0 0%	1 1,0%	0 0%	COPD according to GOLD
Sydykova S. et al. 2008 [49] Kirgizstan (*n* = 125)	139 97,2%	3 2,1%	0 0%	0 0%	0 0%	1 0,7%	0 0%	COPD according to GOLD
Rahaghi et al. 2012 [50] (*n* = 3152) USA	2780 88,2%	124 3,9%	225 7,1%	9 0,28%	10 0,32%	10 0,32%	0 0%	Case-finding in GOLD II-IV sent for Spirometry excluded previously tested patients
This study (*n* = 187) Kazakhstan	181 96.8	3 1,6%	1 0,5%	0 0%	0 0%	0 0%	2 1,1%	COPD according to GOLD

## Conclusions

The present study demonstrates that genetic AATD is present in the Kazakh population. Furthermore, this investigation, performed for the first time with current diagnostic standards in a Kazakh population with COPD, highlights the implication of AATD in the development of COPD. The so-called ‘rare’ AAT alleles may not be as rare as expected. We propose that the rare AAT deficiency variant frequency in Kazakhstan may exceed that observed in this pilot study. This assumption requires verification by case-finding in additional Kazakh cohorts, and could support the want of a target screening for AATD in Kazakhstan, a country where, although this disease has been recognized as rare by Ministry of Health in 2015, a program of detection and specific treatments is still lacking.

## Abbreviations

AAT: Alpha1-antitrypsin
AATD: Alpha1-antitrypsin deficiency
BMI: Body mass index
CAT: COPD assessment test
COPD: Chronic obstructive pulmonary disease
DBS: Dried blood spot
DNA: Deoxyribonucleic acid
ERS: European Respiratory Society
FEV_1_: Forced expiratory volume in one second
FVC: Forced vital capacity
GOLD: Global initiative for chronic obstructive lung disease
IEF: Isoelectrofocusing
kDa: Kilodalton
MRC: Medical research council scale
NE: Neuthrophil elastase
SD: Standard deviation
SERPINA1: Serine (Or Cysteine) proteinase inhibitor
WHO: World Health Organization
